# Diagnosis of chylous abdominal effusions: what is the triglyceride threshold value?

**DOI:** 10.11613/BM.2023.020902

**Published:** 2023-04-15

**Authors:** Bertrand Lefrère, Emmanuel Curis, Randa Bittar, Antoine Levasseur, Pierre Grès, Zoé Guilbert, Belkacem Zenati, Corinne Cherfils, Mehdi Sakka, Dominique Bonnefont-Rousselot

**Affiliations:** 1 Metabolic Biochemistry Department, Pitié-Salpêtrière University Hospital, Assistance Publique - Hôpitaux de Paris, Sorbonne University, Paris, France; 2 UR 7537 BioSTM, Faculty of Pharmacy Paris, Paris City University, Paris, France; 3 Hematology Laboratory, Lariboisière Hospital, Assistance Publique - Hôpitaux de Paris, Paris, France; 4 Sorbonne University, Inserm, UMR_S1166, Paris, France; 5 Faculty of Pharmacy Paris, Paris City University, CNRS, Inserm, UTCBS, Paris, France

**Keywords:** chylous ascites, lipoprotein gel electrophoresis, triglycerides

## Abstract

**Introduction:**

Chylous abdominal effusions are serious complications that can be triggered by various aetiologies. The biochemical diagnosis of chyle leakage in ascites or in peritoneal fluid capsules relies on the detection of chylomicrons. Assaying the fluid’s concentration of triglycerides is still the first-line tool. Given that only one comparative study has sought to quantify the value of the triglyceride assay for diagnosing chylous ascites in humans, our objective was to provide practical triglyceride thresholds.

**Materials and methods:**

We conducted a 9-year, retrospective, single-centre study of adult patients and compared a triglyceride assay with lipoprotein gel electrophoresis for the analysis of 90 non-recurring abdominal effusions (ascites and abdominal collections) of which 65 were chylous.

**Results:**

A triglyceride threshold of 0.4 mmol/L was associated with a sensitivity > 95%, and a threshold of 2.4 mmol/L was associated with a specificity > 95%. According to Youden index, the best threshold was 0.65 mmol/L with a sensitivity of 88 (77-95)%, a specificity of 72 (51-88)%, and, in our series, a positive predictive value of 89 (79-95)% and a negative predictive value of 69 (48-86)%.

**Conclusions:**

In our series, cut-off of 0.4 mmol/L could be used for ruling-out diagnosis of chylous effusions, while cut-off of 2.4 mmol/L could be used for reasonably confirming diagnosis.

## Introduction

The peritoneal fluid (the volume of which ranges from 5 to 100 mL in healthy individuals) is located between the multifunctional, structurally complex mesothelial layers of the body (*1*). Thanks to its humoral and cellular components, this plasma ultrafiltrate constitutes a physiological barrier to infection. Fluid is absorbed from the peritoneal cavity via absorption through lymphatic stomata in the diaphragm and (to a lesser extent) visceral lymphatic pathways and into tissue capillaries (*2*).

Disequilibrium in these absorption processes can trigger ascites, *i.e.* an abnormal accumulation of liquid in the peritoneal cavity. Peritoneal fluids can also accumulate within a capsule. Chylous ascites (probably discovered by Bartolettus) has various congenital or acquired causes: cancer, inflammation, cirrhosis, or trauma (*3*, *4*). Following the puncture or obstruction of the lymphatic network, this effusion can be chylous; the pathological presence of chylomicrons (large lipoproteins consisting mainly of exogenous triglycerides) gives the fluid a milky appearance. However, few epidemiological data on these chylous effusions have been published.

Clinical laboratories must rapidly provide results on biochemical markers, in order to guide the choice of dietary, surgical and/or drug-based treatments (on which consensus guidelines are still lacking) (*4*). A macroscopic, visual assessment of the fluid gives some initial guidance to the clinician but is insufficient *per se*, as a result of potential visual interference: opacity caused by neutrophils in cases of peritonitis, an amber colour due to pancreatitis, a greenish colour due to biliary peritonitis, the presence of cell debris, *etc.* In parallel to a refrigeration test, assaying the peritoneal fluid for triglycerides (TG) (the chylomicron’s main constituent) is still the first-line, rapid, biochemical test for chylous ascites (*5*). The objective of the present study was to provide new, clinically relevant diagnostic cut-offs for the TG assay (relative to the gold standard electrophoretic results) for diagnosing chylous abdominal fluids in adult patients.

## Materials and methods

### Study design

Our single-centre retrospective study covered the period from January 2012 to May 2021. We included abdominal effusion samples collected from adult patients and sent to our laboratory for chyle analysis.

### Subjects

At least 148 samples of ascitic fluid or abdominal drainage fluid (collected after paracentesis and drainage) were sent to the laboratory. In accordance with the French legislation on non-interventional studies of routine clinical practice, the patients were not required to give specific consent but were free to refuse the use of their medical data. All collected data were anonymized before analysis.

To ensure that all samples were independent and to avoid giving too much statistical weight to particular patients, we excluded recurring or multiple samples. Samples whose origin was unclear were also excluded. Of the 90 samples included (most of which were opalescent or milky), 65 (72%) were considered to be chylous (on the basis of the electrophoresis results) and thus confirmed the initial clinical suspicions.

### Methods

Triglyceride and cholesterol concentrations in abdominal liquids were measured in an automated manner by combining three enzyme reactions with a final Trinder reaction and colorimetric detection. Triglycerides are first hydrolysed by lipoprotein lipase. Two auxiliary enzymatic reactions (mediated by a peroxidase) give a detectable quinone-imine. The TG assays were performed with a DiaSys kit (DiaSys Diagnostic Systems GmbH, Holzheim, Germany) on a Konelab 30i system (Thermo Fisher Scientific, France) up until March 2018 (65 samples) and with a TRIGL kit on a Cobas 8000 system (both from Roche Diagnostics GmbH, Mannheim, Germany) thereafter (25 samples). The TG assay gave linear results from 0.0 to 11.3 mmol/L (for DiaSys) and from 0.1 to 10.0 mmol/L (for Roche).

After centrifugation (4437xg for 10 min), the fluid’s appearance was assessed both macroscopically and by measuring the absorbance at 620 nm (Konelab 30i), with the following criteria: 0.001 - 0.080: clear; 0.081 - 0.800: opalescent; 0.800 - 1.000: milky. Lipoproteins were separated electrophoretically with a validated, manual, semiquantitative technique (based on the Hydragel Lipo + Lp(a) K20 kit, Sebia, France), stained with Fat Red reagent (Sudan Red 7B-Aldrich) and revealed with a Hyrys2 system (Sebia) (*6*). A sample was defined as chylous when material in the gel’s loading well was stained (highlighting the presence of chylomicrons unable to migrate).

### Statistical analysis

Our analysis was based on the area under the receiver operating characteristic (ROC) curve and its 95% confidence interval (95%CI), calculated with a bootstrap method. For a given threshold, the sensitivity, specificity, positive predictive value, and negative predictive value were quoted with their exact 95%CIs (calculated using the binomial law). “Best” threshold was defined as the threshold that maximizes the Youden index, and its confidence interval obtained by bootstrap.

Since continuous variables studied here were not expected to have a Gaussian distribution (especially TG and cholesterol concentrations), comparison between chylous and non-chylous were preplanned to be made using non-parametric methods (Wilcoxon-Mann-Whitney). This avoids both multiple testing issues and unknown control of global Type-I error due to hierarchical testing.

Samples from patients with chylous *vs.* non-chylous samples were compared using Fisher’s exact test (for qualitative data) or the Wilcoxon-Mann-Whitney test (for quantitative data). Since the analysis was descriptive, P-values were not corrected for multiple testing. All statistical analyses were performed using R software (version 4.0.2) and the pROC package (version 1.18.0) (*7*, *8*).

## Results

**Figure 1:**
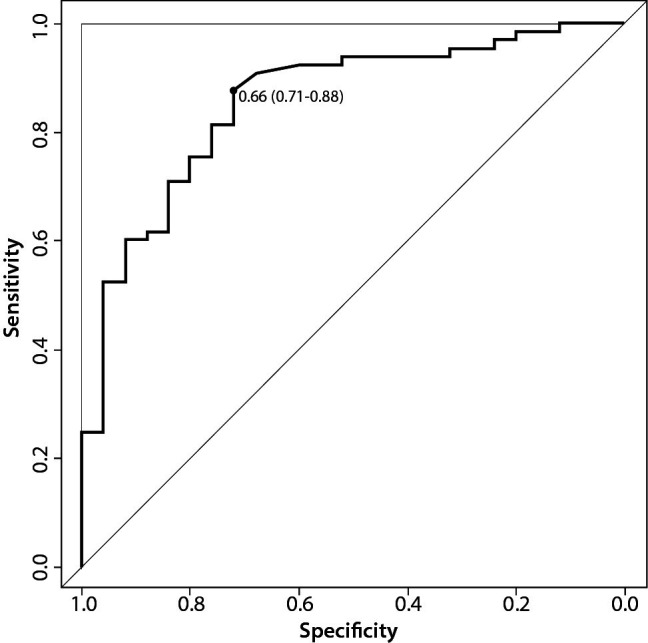
The ROC curve for prediction of the presence of chyle in ascites liquid, based on the triglyceride concentration.

**Figure 2:**
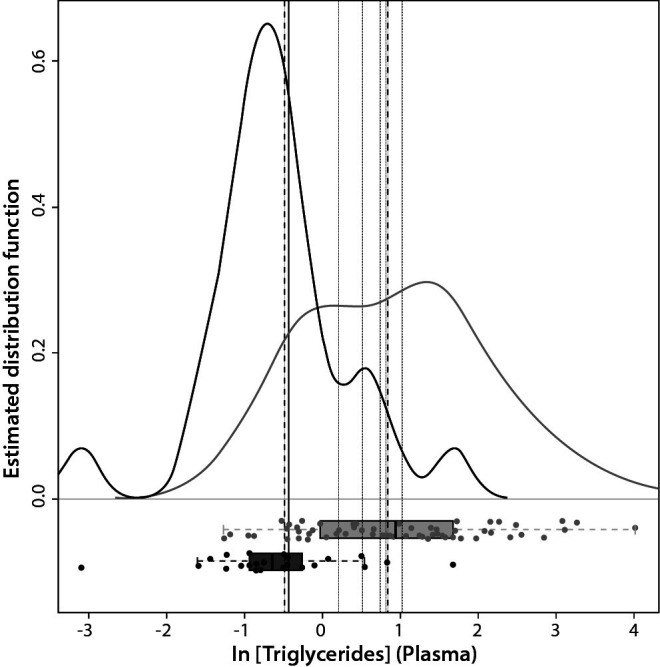
Comparison of the triglyceride distribution in chylous samples (in grey) *vs.* non-chylous samples (in black). Black vertical lines show the optimal threshold according to Youden’s index (the continuous line) and its 95% confidence interval (the dashed line). The vertical dotted lines show the thresholds reported by Staat *et al.* (1.2 mmol/L) and Thalers *et al.* (2.1 mmol/L).

The patients’ epidemiological and laboratory characteristics are summarized in Table 1. Discriminating power of TG concentration, to distinguish chylous and non-chylous samples, is summarized by the ROC curve (Figure 1). The area under this ROC curve was 0.85 (95%CI: 0.77-0.94), significantly greater than 0.5. The TG concentrations in the chylous and non-chylous samples are compared in Figure 2.

**Table 1:** Demographic and biochemical results

	**Chylous samples (N = 65)**	**Non-chylous samples (N = 25)**	**P**
Gender, males (N, proportion)	34 (0.52)	16 (0.64)	0.352
Age, years	68 (56-73)	68 (42-77)	0.715
Sample appearance			
clear	4	14	
opalescent	9	9	< 0.001
milky	32	2	
TG (mmol/L)	2.5 (1.0-5.4)	0.4 (0.4-0.8)	< 0.001
Cholesterol (mmol/L)	1.3 (0.6-1.8)	0.9 (0.4-1.9)	0.337
TG – triglycerides. Continuous variables are expressed as the median (interquartile range). P < 0.05 was considered statistically significant.			

As shown in Table 2, a TG threshold of 0.4 mmol/L gave a sensitivity of > 95%, and a threshold of 2.4 mmol/L gave a specificity of > 95%. According to the Youden index, the best threshold was 0.65 mmol/L (95%CI 0.6-2.4 mmol/L), associated with a sensitivity of 88 (77-95)%, a specificity of 72 (51-88)%, a positive predictive value of 89 (79-95)% and a negative predictive value of 69 (48-86)%. In line with Thaler *et al.*’s conclusions, the triglyceride/cholesterol ratio (area under the curve (95%CI): 0.79 (0.68-0.90) did not provide additional diagnostic value and might even decrease it.

**Table 2:** Selected ROC curve data for decisional cut-offs

**TG threshold (mmol/L)**	**True positive (N)**	**False positive (N)**	**False negative (N)**	**True negative (N)**	**Sensitivity (%)**	**Specificity (%)**	**Predictive positive value (%)**	**Predictive negative value (%)**
0.4	62	17	3	6	95	32	79	73
0.65	57	7	8	18	88	72	89	69
2.4	34	1	31	24	52	96	97	44
TG – triglycerides.								

## Discussion

To the best of our knowledge, there is only one comparative study in the indexed medical literature on the TG assay’s value in diagnosing chylous ascites in humans (*9*). Thaler’s cut-offs for good sensitivity (> 95%), good specificity (> 95%), and for Youden index are, respectively, 1.7 mmol/L, 2.8 mmol/L, 2.1 mmol/L. We found quite a different threshold for good sensitivity (> 95%) (*9*). Indeed, our threshold for good sensitivity was about four times lower than that reported by Thaler *et al.* In contrast, our threshold for good specificity (> 95%) was quite close to Thaler *et al.*’s value. Although the two studies had similar diagnostic objectives, the respective datasets probably differed in several respects. With regard to analytical variables, we do not know whether the respective assay methods were correlated. One can also hypothesize that a low proportion of the electrophoresis results could be false positives (*10*). With regard to clinical variables, there may have been inter-study differences in recruitment (Thaler *et al.* included more than one sample for a given patient, in some instances), aetiologies, and prescribing habits (*e.g.* prescription of the electrophoresis assay). The presence of inter-study differences in TG thresholds suggests that second-line techniques (such as gel lipoprotein electrophoresis or the observation of stained samples under the microscope) should be used more frequently. Relative to our study, Thaler *et al.* used the same R package and a similar statistical approach but did not report a CI for the proposed threshold. Our 95%CI is broad and includes the threshold value that Thaler *et al.* determined from the Youden index. The inter-study difference might be not significant and might reflect inter-individual variability and the small size of both datasets.

It might be possible to choose an appropriate TG threshold for each effusion site; the values in the literature are not clear, and original citations are often lacking. The threshold of 1.2 mmol/L might correspond to Staats *et al.*’s historical conclusions from a study that focused on chylothorax (*11*). The results of Thaler *et al.*’s retrospective study emphasized the relatively low diagnostic value of cholesterol assays and suggested the following TG threshold values (relative to the gold standard lipoprotein gel electrophoresis): 2.1 mmol/L when maximizing Youden index (with a sensitivity of 87% and a specificity of 89%), 1.7 mmol/L for a sensitivity greater than 95%, and 2.8 mmol/L for a specificity greater than 95% (*9*). However, these conclusions were challenged by Miserach *et al.*, who reported that the thresholds lacked sensitivity in their validation of a large retrospective cohort (*12*). The important overlap between TG values in chylous and non-chylous effusion observed in our study explains our large CI on the threshold, and suggests that defining a single threshold will hardly give both satisfactory sensitivity and specificity. In our series, cut-offs of 0.4 mmol/L and 2.4 mmol/L could be used, respectively, for infirming or confirming the hypothesis of a chylous effusion.

Our study had several limitations. Firstly, it was a retrospective study of samples from adult patients only. Secondly, the source of some of the effusion fluid samples sent to the laboratory was sometimes not specified, and so we had to assign this variable retrospectively. Thirdly, we did not check for potential blood contamination at the time of sampling. Fourthly, we did not document the type of drainage used (which can influence the flow rate), the time that the sample had spent at room temperature prior to the assay (which can affect the TG stability), or the patient’s nutritional status (which can influence chylomicron generation). Fifthly, we did not distinguish between the various aetiologies of chylous ascites and abdominal chyle leakage. Sixthly, recruitment bias might have affected the “control” fluid samples, *i.e.* those lacking chylomicrons in the lipoprotein gel electrophoresis. As all fluid samples were initially sent to the laboratory to be checked for chylomicrons, the predictive values obtained with opaque or milky samples might not be easily extrapolated to series of samples with less stringent inclusion criteria. Lastly, the TG content was not corrected for the glycerol concentration.

Analysis of a more direct biomarker of chylomicrons, *e.g.* apolipoprotein apoB48 (for research purpose), could be interesting (*13*). The conclusions of the second comparative study of the diagnostic TG threshold (a first-line biomarker, in the absence of lipoprotein electrophoresis) for chylous abdominal effusions must now be confirmed in studies with a more refined design, given all the possible sources of bias mentioned above. It would also be interesting to reconsider these questions in paediatric/neonatal populations, who differ from adults with regard to lipid metabolism.
